# Indirect Effect of a Transgenic Wheat on Aphids through Enhanced Powdery Mildew Resistance

**DOI:** 10.1371/journal.pone.0046333

**Published:** 2012-10-08

**Authors:** Simone von Burg, Fernando Álvarez-Alfageme, Jörg Romeis

**Affiliations:** 1 Institute of Evolutionary Biology and Environmental Studies, University of Zurich, Zurich, Switzerland; 2 Agroscope Reckenholz-Tänikon Research Station ART, Zurich, Switzerland; Nanjing Agricultural University, China

## Abstract

In agricultural ecosystems, arthropod herbivores and fungal pathogens are likely to colonise the same plant and may therefore affect each other directly or indirectly. The fungus that causes powdery mildew (*Blumeria graminis tritici*) and cereal aphids are important pests of wheat but interactions between them have seldom been investigated. We studied the effects of powdery mildew of wheat on two cereal aphid species, *Metopolophium dirhodum* and *Rhopalosiphum padi.* We hypothesized that aphid number and size will be smaller on powdery mildew-infected plants than on non-infected plants. In a first experiment we used six commercially available wheat varieties whereas in the second experiment we used a genetically modified (GM) mildew-resistant wheat line and its non-transgenic sister line. Because the two lines differed only in the presence of the transgene and in powdery mildew resistance, experiment 2 avoided the confounding effect of variety. In both experiments, the number of *M. dirhodum* but not of *R. padi* was reduced by powdery mildew infection. Transgenic mildew-resistant lines therefore harboured bigger aphid populations than the non-transgenic lines. For both aphid species individual size was mostly influenced by aphid number. Our results indicate that plants that are protected from a particular pest (powdery mildew) became more favourable for another pest (aphids).

## Introduction

In agro-ecosystems, both insect herbivores and pathogenic fungi are abundant and are likely to colonise the same plant. Both depend on plant tissue, and each may alter the conditions for the other. Interactions can be direct, indirect (plant-mediated) or both. Direct interactions include, for instance, feeding relationships. Occasionally, herbivores feed upon fungal pathogens or fungus-infected plant tissue [1], [2]. In contrast, plant tissue damaged by herbivore feeding facilitates the entrance of fungal pathogens [3]. Insects can also act as dispersing vectors for fungi [4]. Indirect, plant-mediated interactions result from modifications in the allocation of plant metabolites or through plant defence mechanisms and can be caused by the herbivore or the pathogen [2], [5], [6]. Either party can thus alter the suitability and quality of the host plant for the other, and often does so in a negative way [1].

Aphids are important herbivore pests [7], [8]. As strict herbivores, aphids depend on plant tissue and phloem-sap throughout their life and, therefore, are very sensitive to metabolic and physiological changes in their host plants. Their performance can depend on phloem-sap composition [9]–[12] as well as on secondary plant metabolites [13]–[16].

Many studies have shown that the application of fungicides to crops such as tomato, potato, and alfalfa generally result in increased aphid populations [17]–[21]. Most of these studies argue that the fungicides interfere with entomopathogenic fungi, leading to decreased aphid mortality or other direct fungicide-related effects. Our own observations from a semi-field experiment, however, indicated that transgenic wheat (*Triticum aestivum* Linné) resistant to powdery mildew [*Blumeria graminis* (DC.) Speer var. *tritici*] had higher cereal aphid populations than non-transgenic, susceptible wheat lines [22], [23] even though no fungicides were applied. This suggested that fungicide-independent, plant-mediated effects were influencing aphid populations. In this study, our objective was to confirm this effect and investigate the interactions between powdery mildew of wheat and two common cereal aphid species.

The two cereal aphid species, *Metopolophium dirhodum* Walker and *Rhopalosiphum padi* Linné, are globally distributed generalists. Both are cyclical parthenogens that alternate between their primary and secondary host plants, which include wheat and other major cereals. The causal agent of wheat powdery mildew, is an obligate, biotrophic fungus that is widely distributed throughout the world. It especially thrives in cool, humid regions and, if infection levels are high, it reduces yield [8], [24]. Powdery mildew is easy to detect because the mycelium forms a white, fluffy layer on the leaves.

In no-choice laboratory population experiments, we measured aphid numbers and individual aphid size on powdery mildew-infected and uninfected plants. Based on the previous findings reported by von Burg et al. [22] and Álvarez-Alfageme et al. [23], we hypothesized that aphid number and body size will be smaller on wheat infected with powdery mildew than on healthy plants. We tested this hypothesis in two experiments. Experiment 1 used a range of conventional wheat varieties that were inoculated or not with powdery mildew. Experiment 2 avoided the effects caused by using different wheat varieties by using a genetically modified (GM) powdery mildew-resistant wheat line and its corresponding, non-segregant sister line. These two lines, which differ in susceptibility to powdery mildew, are genetically identical except for the presence or absence of the transgene. To exclude unintended effects on the aphids caused by the genetic modification, experiment 2 also used two powdery mildew isolates that differed in virulence; one of the isolates could break the resistance of the GM line (virulent strain V) and the other could not (avirulent strain A).

## Materials and Methods

### Insect and Plant Material

Laboratory cultures of *M. dirhodum* and *R. padi* were founded from individuals collected from several wheat fields around Zurich (Switzerland) during summer 2007 and 2008, respectively. The cultures were reared on the winter wheat variety Camedo and kept in climate chambers at 22°C, 80% r.h., and a 16∶8 h light:dark regime.

In experiment 1, we used six conventional wheat varieties: Bobwhite, Casana, Fiorina, Frisal, Rubli, and Toronit. Seeds were provided by the Agroscope Reckenholz-Tänikon Research Station ART. In experiment 2, we used an experimental GM wheat line, Pm3b*#1*, and its respective control line, Sb*#1*. The GM line Pm3b*#1* was engineered from Bobwhite and carries the transgene *Pm3b* of wheat, which confers race-specific resistance to powdery mildew [25], [26]. As noted earlier, the corresponding non-segregant sister line Sb*#1* is genetically identical except for the absence of the transgene, which makes the line susceptible to powdery mildew. Pm3b*#1* and Sb*#1* plants are further described in Zeller et al. [27] and Brunner et al. [28] and have shown an enhanced resistance against powdery mildew under protected glasshouse, semi-field, and field conditions [23], [27], [28]. The seeds of both experimental lines were provided by the Institute of Plant Biology (University of Zurich).

### Powdery Mildew Strains

We used two strains of the wheat powdery fungus *B. graminis* f. sp. *tritici*. Strain No.96229 is avirulent on plants with the *Pm3b* transgene [29]. Strain No.98229 is virulent on plants with the *Pm3b* transgene [26]. Both mildew strains were obtained from a collection at the Institute of Plant Biology (University of Zurich) and are hereafter referred to as strain A for avirulent and strain V for virulent.

### Experiments

Experiment 1 included the six commercially available wheat varieties that were inoculated or not inoculated with mildew strain A and with one of the two aphid species. There were 10 replicates per combination of factors, resulting in a total of 240 plants: 2 levels of fungus inoculation × 2 aphid species × 6 wheat varieties × 10 replicates. Because of its size, experiment 1 was conducted in two temporal blocks of five replicates per block.

In experiment 2 we included the wheat lines Pm3b*#1* and Sb*#1* that were inoculated with mildew strains A or V or were not inoculated. Plants were then also infested with one of the two aphid species. There were 10 replicates per combination of factors, resulting in a total of 120 plants: 3 levels of fungus inoculation (A, V, or control) × 2 aphid species × 2 wheat varieties × 10 replicates.

The following procedures were used for both experiments, except as noted. The plants were individually grown in 3-L pots (3 seeds were sown per pot) under constant conditions (22°C, 60% r.h., 16∶8 h light dark) in the greenhouse. The pots contained composted soil, and each was fertilized with 3 g of Osmocote Exact slow release granules at sowing (N15 : P9 : K9 : Mg3, Scotts Italia SRL, Italy). In addition, plants were fertilized weekly with 150 ml of a 0.2% aqueous solution of Vegesan Standard (80 g N, 70 g P_2_O_5_, 80 g K_2_O, Hauert HBG Dünger AG, Grossaffoltern, Switzerland). Plants were watered as required. Ten days after sowing the plants were thinned out and only one plant was left per pot. Three weeks after sowing, the seedlings were moved into separate greenhouse compartments (with the same conditions) where they were inoculated with the fungus. For experiment 1, half the plants were inoculated with mildew strain A and half the plants were not inoculated. In experiment 2, one-third of the plants were inoculated with strain A, one-third were inoculated with strain V, and one-third served as non-inoculated controls. Because *B. graminis* f. sp. *tritici* is an obligate biotrophic pathogen, *in vitro* cultivation is not possible. The inoculum was therefore produced by infecting the susceptible wheat variety Kanzler. These infected host plants were then equally brushed over the experimental wheat lines, which distributed conidia rapidly and fairly uniformly [30].

After inoculation, all plants were individually caged with plastic bags (Egli Plastic AG, Dällikon, Switzerland) to prevent future aphid and mildew cross-contamination. Inoculated and non-inoculated plants were kept separately for the next two weeks, during which symptoms of mildew infection became evident. After these two weeks, the plants were equally distributed in two greenhouse compartments so that each one contained an equal number of replicates per treatment combination.

In the next step, we transferred ten 1^st^ to 2^nd^ instar nymphs of either *M. dirhodum* or *R. padi* to each plant using a sucking tube. The aphids were then left for three weeks to establish a population corresponding to about three aphid generations. After this three-week period, the experiment was stopped. Mildew infection intensity was determined on the whole canopy using the Cobb’s scale, which ranges from 0 to 9 [31]. The plants were cut just above the soil line, and the tops were bagged and stored in a −80°C freezer for further analysis. Subsequently, the aphids on each plant were counted, and the hind tibia length from five randomly selected adult aphids per plant was measured with a binocular and an ocular micrometer (Zeiss, Feldbach, Switzerland). Plant above-ground biomass was determined by drying the plants at 80°C for 24 h and weighing them to the nearest 0.01 g (Mettler Toledo, Greifensee, Switzerland). Because pathogen infection can lead to changes in nitrogen tissue concentrations [32], we determined the C:N ratio of five randomly chosen plants per wheat line and mildew infection treatment to obtain an estimate of changes in plant metabolites. The C:N ratio was assessed by thermal combustion of about 3 g of dried and powdered leaf material (Leco CHNS-932 Elemental Analyzer, Leco Corporation, St. Joseph, MI, USA).

### Statistical Analyses

For both experiments, the data for the two aphid species were analysed separately. Aphid numbers were square-root transformed to meet model assumptions, whereas data for aphid size were analysed untransformed. For the analysis of aphid number we included plant biomass as a covariable. For analysis of aphid size we included aphid number as a covariable. In both experiments, some replicates were lost because of aphid cross-contaminations or infections with an entomopathogenic fungus. Therefore, degrees of freedom did not match those predicted by the original design.

Data from experiment 1 were analysed using a linear mixed-effects (LME) model based on *F* statistics. The model included fungus inoculation, wheat variety, and their two-way interaction as fixed effects and temporal block and greenhouse compartment as random effects. For experiment 2 we also used an LME but with different *a priori* contrasts. The first contrast compared the three levels of fungus inoculation (i.e., not inoculated, inoculated with strain A, and inoculated with strain V). The other three contrasts compared GM vs. non-GM plants within each level of fungus inoculation.

For both experiments, C:N ratios were arcsine transformed and analysed using LME. Fungus inoculation, GM, and their interaction were fixed effects. All analyses were done with the statistical software R (R development core team) or GenStat (VSN International Ltd.).

## Results

### Experiment 1

**Figure 1:**
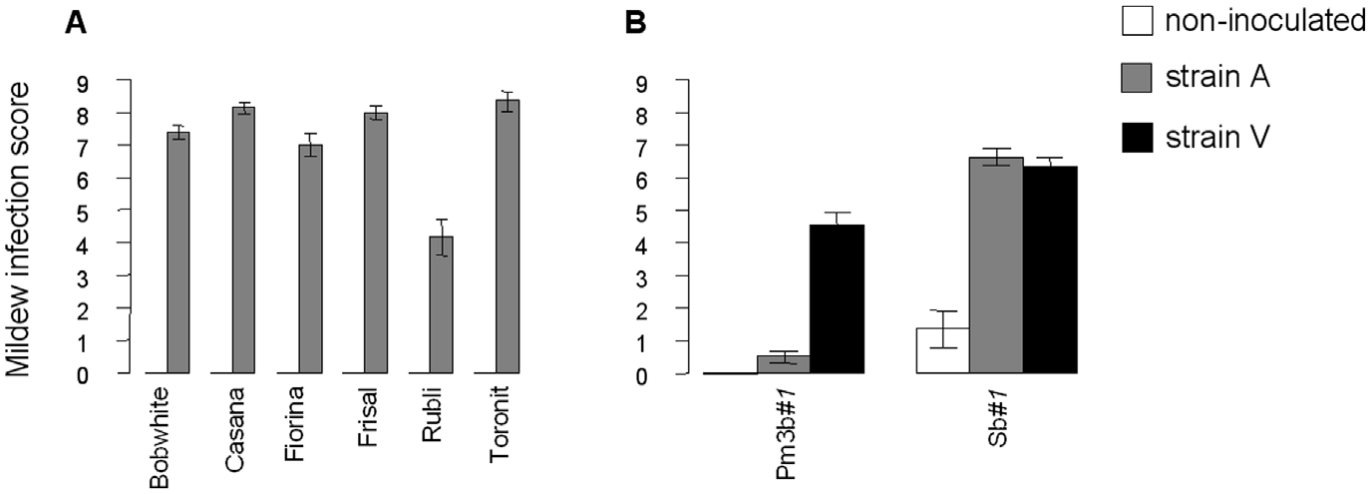
Mildew infection levels in the two experiments. Mildew infection levels (according to Cobbs’ scale) as affected by six commercially available wheat line in experiment 1 (A) or by wheat line (transgenic Pm3b*#1* vs. non-transgenic Sb*#1*) and mildew strain (A, V, and non-inoculated control) in experiment 2 (B). Values are means ± SEM. In experiment 1, all plants were inoculated with strain A or were non-inoculated, and all non-inoculated plants remained healthy (white bars do not appear).

The inoculated plants in the experiment involving six commercial wheat varieties had severe mildew infection symptoms, whereas the non-inoculated plants remained healthy (Fig. 1B). The wheat varieties differed significantly in their infection levels (*F_5,224_* = 20.25, *p*<0.001).

Mean C:N ratios ranged from 7.45 (±0.76; SE) to 9.59 (±0.85) and were not significantly affected by the fungus treatment or wheat line.

**Figure 2:**
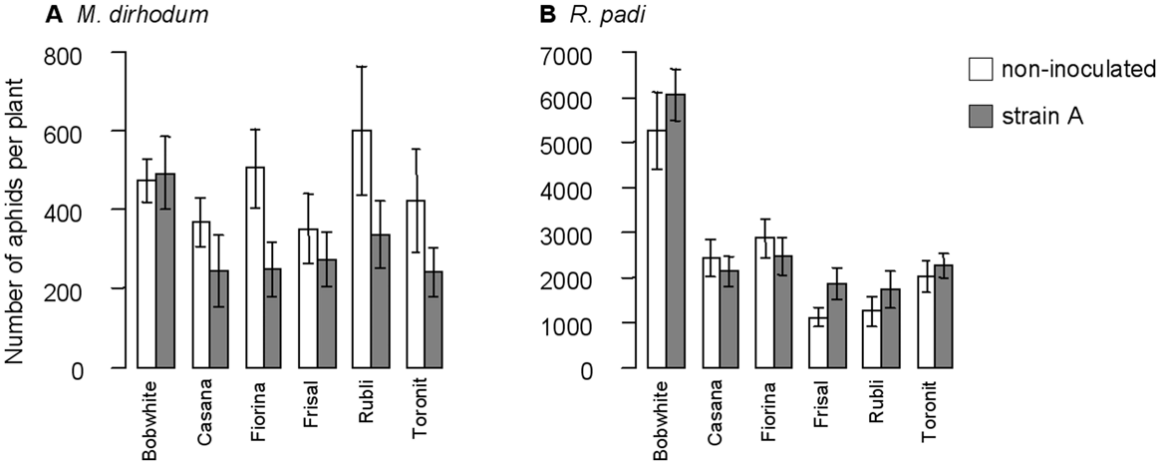
Aphid numbers on the different wheat lines and treatments in experiment 1. Effect of six wheat varieties and powdery mildew inoculation on numbers of *M. dirhodum* (A) and *R. padi* (B) per plant in experiment 1. Values are means ± SEM for non-inoculated plants (white bars) and for plants inoculated with mildew strain A (grey bars).

Numbers of *M. dirhodum* were significantly smaller on the powdery mildew-infected plants than on healthy plants (*F_1,74_* = 12.10, *p*<0.001) whereas *R. padi* numbers were not affected by fungus inoculation (Fig. 2). *Rhopalosiphum padi* numbers were affected by wheat variety (*F_5,105_* = 14.43, *p*<0.001) and plant biomass (*F_1,105_* = 47.04, *p*<0.001), neither of which influenced *M. dirhodum*.

The tibia length of adults from both aphid species were smaller on the infected plants than on the non-infected plants (Table S1), although the effect was only marginally significant for *R. padi* (*M. dirhodum*: *F_1,72_* = 43.22, *p*<0.001; *R. padi*: *F_1,83_* = 3.61, *p* = 0.061). The size of *M. dirhodum* was marginally influenced by wheat variety (*F_1,72_* = 2.23, *p* = 0.060) and was positively associated with population size (*F_1,72_* = 29.88, *p*<0.001). For *R. padi* size, the interaction between fungus inoculation and wheat variety was significant (*F_1,83_* = 2.32, *p* = 0.050).

### Experiment 2

In the experiment with the transgenic wheat plants, the three fungus treatments (non-inoculated, inoculated with strain A, and inoculated with strain V) resulted in the expected infections of the two wheat lines (Fig. 1B). Both mildew strains caused severe symptoms on the non-transformed control line Sb*#1,* whereas the non-inoculated Sb*#1* plants remained healthy. The transgenic Pm3b*#1* line had no mildew symptoms when inoculated with strain A or when non-inoculated but had significant infection levels when inoculated with strain V. There were some mildew cross-infections, but because infection levels were very low, we did not exclude these plants from the analysis. Mean C:N ratios ranged from 7.35 (±0.76) to 8.29 (±0.34) and were not significantly affected by the fungus treatment or wheat line.

**Figure 3:**
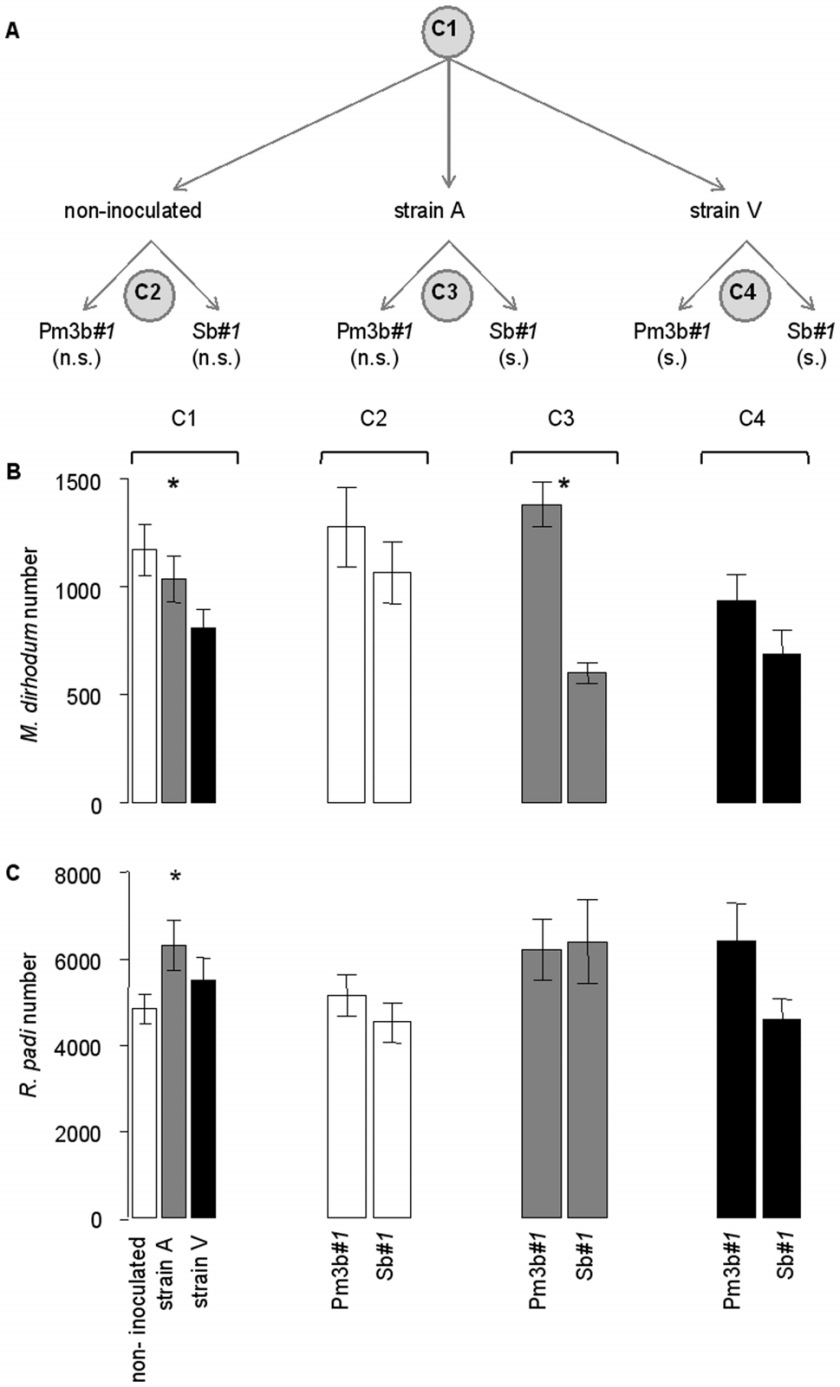
Analysed contrasts and corresponding aphid numbers on different wheat lines and treatments in experiment 2. Effects of two wheat lines (transgenic Pm3b*#1* vs. non-transgenic Sb*#1*) and powdery mildew inoculation on aphid numbers in experiment 2. The flow-diagram in (A) describes the four orthogonal contrasts (indicated by C1–C4 in grey circles) and whether the two lines exhibited powdery mildew symptoms when not inoculated with the fungus or when inoculated with strain A or V: s. and n.s. in parentheses in (A) indicate that the plants did or did not exhibit powdery mildew symptoms, respectively. Contrasts C1–4 concern the effects of wheat line and powdery mildew strain on numbers of *M. dirhodum* (B) and *R. padi* (C) per plant. Values in (B) and (C) are means ± SEM for non-inoculated plants (white bars) and for plants inoculated with strain A (grey bars) and strain V (black bars). Asterisks indicate significant differences (* *p*<0.05, *** *p*<0.001).

The orthogonal *a priori* contrasts used for analysis of experiment 2 are described in Figure 3A. Fungus inoculation significantly affected *M. dirhodum* numbers. Numbers were largest on the non-inoculated plants and smallest on plants inoculated with strain V (*F_2,45_* = 8.48, *p*<0.001) (Fig. 3B, C1). As expected, the plants inoculated with strain A had intermediate *M. dirhodum* numbers because these numbers were from infected Sb*#1* plants and resistant, symptomless Pm3b*#1* plants. Within the three fungus treatments, *M. dirhodum* numbers did not differ between non-inoculated Pm3b*#1* and non-inoculated Sb*#1* plants (Fig. 3B, C2), or between the Pm3b*#1* and Sb*#1* plants inoculated with mildew strain V (Fig. 3BB, C4). When plants were inoculated with mildew strain A, in contrast, *M. dirhodum* numbers were larger on the resistant Pm3b*#1* plants than on the susceptible Sb*#1* plants (*F_1,45_* = 8.36, *p* = 0.006) (Fig. 3B, C3). *Rhopalosiphum padi* numbers in experiment 2 were largest on the plants inoculated with strain A (*F_2,52_* = 4.06, *p* = 0.023) (Fig. 1C, C1) The other contrasts were not significant (Fig. 1C, C2–C4). Plant biomass had a significant and positive effect on both aphid species in experiment 2 (*M. dirhodum*: *F_1,48_* = 33.10, *p*<0.001; *R. padi*: *F_1,52_* = 95.66, *p*<0.001).

The tibia length of *M. dirhodum* adults was unaffected by fungus inoculation or wheat line (Table S2). However, *M. dirhodum* size was again positively related with aphid number (*F_1,48_* = 11.69, *p*<0.001). *Rhopalosiphum padi* were larger on the non-inoculated non-transgenic Sb*#1* plants than on the non-inoculated transgenic Pm3b*#1* plants (*F_1,52_* = 4.67, *p* = 0.035). Further, *R. padi* size tended to decline as *R. padi* numbers increased (*F_1,52_* = 3.91, *p* = 0.053).

## Discussion

In this study, we investigated how two aphid species, *M. dirhodum* and *R. padi*, were affected by powdery mildew of wheat caused by the fungus *B. graminis* f. sp. *tritici*. To do this, we used different wheat lines including an experimental GM line and measured aphid number and size.

The two aphid species reacted quite differently to the presence of powdery mildew. For *M. dirhodum,* we confirmed our initial hypothesis regarding effects on aphid number, which was that numbers would be smaller on powdery mildew-infected plants irrespective of the wheat variety or the presence of the transgene. The effects of powdery mildew on *M. dirhodum* size were not consistent in the two experiments. In both experiments, however, was *M. dirhodum* size positively associated with aphid number, which would again be consistent with the hypothesis, given that *M. dirhodum* numbers were smaller on infected plants. *Rhopalosiphum padi* numbers, in contrast, were mostly unaffected by powdery mildew but were affected by wheat variety and plant biomass. In experiment 2, which used a GM line and its non-GM sister line, *R. padi* numbers were unexpectedly larger on plants inoculated with mildew strain A; we cannot explain this result because it cannot be related to wheat line or to powdery mildew infection according to our analyses.

What are the possible mechanisms that cause *M. dirhodum* numbers to be smaller on powdery mildew-infected plants? We first consider non-physiological effects. Higher aphid number on healthy than on diseased plants could occur if aphid preferably colonise pathogen-free plants; in our experiments, however, all plants were inoculated with 10 aphids, i.e., aphids were not allowed to choose their host plant. Other studies that reported greater aphid numbers on healthy than on diseased plants used fungicide to obtain different levels of disease, and fungicide might have directly affected the aphids in these studies [18]–[21]. In the current study, *M. dirhodum* suppression cannot be explained by effects of fungicides because no fungicides were applied. The mycelium of the mildew fungus might physically suppress *M. dirhodum* feeding because it forms a fluffy layer that covers the leave surface and that might hinder stylet penetration. Pathogen infection might also cause cell-wall thickening or other changes in the consistency of the plant epidermis [33], [34] that could hinder stylet penetration. These are, however, unlikely explanations for the aphid suppression observed in our study because mycelia and cell-wall thickening are usually local effects; when confronted with fungal colonies, aphids could presumably move to a more suitable part of the plant. Also, suppression of aphid feeding resulting from mycelia or cell-wall thickening fails to explain why only one of the two aphid species was affected. It is thus more likely that aphid suppression resulted from changes in plant physiology.

Fungal pathogens can change the allocation of plant metabolites and induce plant defence mechanisms [35] which might change the nutrition provided to aphids by its host plant. Changes in the carbohydrate composition of phloem sap could explain the different reactions of the two aphid species. Sucrose is the dominant sugar in phloem sap [36] and Pescod, Quick & Douglas [37] documented that the response of aphids to altered sucrose levels in the phloem sap is species-specific, i.e., a reduction in sucrose suppressed the population growth of some aphid species but not the others. This could explain the species-specific reaction in our study. To obtain a rough estimate about changes in plant metabolites, we determined the C:N ratio of the different wheat lines in both experiments but did not find any treatment effects. However, the C:N ratio might be insufficiently sensitive for detecting changes in phloem sap composition, and finer methods such as phloem sap or aphid honeydew-analyses are needed to confirm compositional phloem-sap changes and their influence on the two aphid species.

A physiological explanation for the suppression of *M. dirhodum* numbers by powdery mildew in the current study is also suggested by the positive relationship between *M. dirhodum* numbers and size. Previous research with the aphid *Myzus persicae* demonstrated that life-history traits, such as individual size, development time, and fecundity, are all positively correlated [38]; we have documented similar relationships in a life-table experiment with *M. dirhodum* (unpublished data, based on von Burg et al., Müller & Romeis [39]). It is therefore likely that the larger *M. dirhodum* numbers on the healthy plants are due to a cumulative effect of shorter development times and higher fecundity, all traits that were unaffected in the life-table experiment using the same Pm3b-transgenic wheat lines but without mildew infection [39]. In contrast to *M. dirhodum, R. padi* body size and numbers were negatively associated but only when numbers were really high, as they were in experiment 2. Thus, the negative relationship between aphid size and aphid number for *R. padi* was probably due to crowding. Summarizing, we can say that healthy plants provide a better habitat and better nutritional quality than diseased plants for *M. dirhodum*.

Overall, we showed that the control of one agricultural pest resulted in healthier plants that in turn became more favourable for another potential pest. That the release from resource competition can benefit other plant pests is a common phenomenon [2], [5], [40] that has been reported to contribute to emerging pest problems for example in insect-resistant transgenic cotton [41]. It follows that crop protection targeting a particular pest, whether that protection is based on genetic modification or conventional control methods, should always account for the possibility of increased attack by other pests.

## Supporting Information

Table S1 **Aphids’ tibia lenght on the different wheat lines and treatments in experiment 1.** Effect of six wheat varieties and powdery mildew inoculation (see Fig. 1A) on the tibia lenght of *Metopolophium dirhodum* and *Rhopalosiphum padi* adults in experiment 1. Values are means (mm) ± SEM.
(DOCX)

Table S2 **Aphids’ tibia lenght on the different wheat lines and treatments in experiment 2.** Effect of two wheat lines (transgenic Pm3b*#1* vs. non-transgenic Sb*#1*) and powdery mildew inoculation on the tibia lenght of *Metopolophium dirhodum* and *Rhopalosiphum padi* adults in experiment 1. Values are means (mm) ± SEM.
(DOCX)
