# Supplementary material for: Indirect Effect of a Transgenic Wheat on Aphids through Enhanced Powdery Mildew Resistance
Source: PLoS One. 2012 Oct 8;7(10):e46333. doi: 10.1371/journal.pone.0046333 (PMC3466243; doi:10.1371/journal.pone.0046333)
Supplement: Table S1 — Aphids’ tibia lenght on the different wheat lines and treatments in experiment 1. Effect of six wheat varieties and powdery mildew inoculation (see Fig. 1A) on the tibia lenght of Metopolophium dirhodum and Rhopalosiphum padi adults in experiment 1. Values are means (mm) ± SEM. (DOCX) [file pone.0046333.s001.docx]

|  | *Metopolophium dirhodum* | | *Rhopalosiphum padi* | |
| --- | --- | --- | --- | --- |
| Wheat line | Non-inoculated | Strain A | Non-inoculated | Strain A |
| Bobwhite | 1.67 ± 0.053 | 1.56 ± 0.026 | 0.78 ± 0.027 | 0.79 ± 0.037 |
| Casana | 1.62 ± 0.069 | 1.43 ± 0.029 | 0.79 ± 0.020 | 0.76 ± 0.036 |
| Fiorina | 1.57 ± 0.085 | 1.38 ± 0.028 | 0.80 ± 0.029 | 0.77 ± 0.042 |
| Frisal | 1.67 ± 0.055 | 1.51 ± 0.038 | 0.86 ± 0.024 | 0.77 ± 0.049 |
| Rubli | 1.64 ± 0.032 | 1.58 ± 0.077 | 0.86 ± 0.038 | 0.74 ± 0.025 |
| Toronit | 1.70 ± 0.045 | 1.48 ± 0.040 | 0.83 ± 0.032 | 0.81 ± 0.038 |
