# Supplementary material for: Indirect Effect of a Transgenic Wheat on Aphids through Enhanced Powdery Mildew Resistance
Source: PLoS One. 2012 Oct 8;7(10):e46333. doi: 10.1371/journal.pone.0046333 (PMC3466243; doi:10.1371/journal.pone.0046333)
Supplement: Table S2 — Aphids’ tibia lenght on the different wheat lines and treatments in experiment 2. Effect of two wheat lines (transgenic Pm3b#1 vs. non-transgenic Sb#1) and powdery mildew inoculation on the tibia lenght of Metopolophium dirhodum and Rhopalosiphum padi adults in experiment 1. Values are means (mm) ± SEM. (DOCX) [file pone.0046333.s002.docx]

|  | *Metopolophium dirhodum* | | | *Rhopalosiphum padi* | | |
| --- | --- | --- | --- | --- | --- | --- |
| Wheat line | Non-inoculated | Strain A | Strain V | Non-inoculated | Strain A | Strain V |
| Pm3b#1 | 1.33 ± 0.014 | 1.34 ± 0.027 | 1.33 ± 0.028 | 0.67 ± 0.016 | 0.72 ± 0.012 | 0.71 ± 0.018 |
| Sb#1 | 1.34 ± 0.024 | 1.26 ± 0.034 | 1.28 ± 0.015 | 0.71 ± 0.011 | 0.71 ± 0.022 | 0.72 ± 0.020 |
